# Right low abdominal pain (RLAP): a new signal in food allergy

**DOI:** 10.1186/1939-4551-8-S1-A280

**Published:** 2015-04-08

**Authors:** Isaac Azevedo Tenorio, Aderbal Sabra, Selma Sabra

**Affiliations:** 1 Unigranrio, Brazil

## Objectives and study

The objective of this study is to describe the pain in the right low abdominal area, i.e. , in the right iliac fossa, as a signal and symptom findings in the physical examination in patients with food allergy.

## Methods

We studied the data of the physical examination of the right low abdômen área of 355 patients with food allergy, retrospective using the medical data from the medical records of the Brazilian Society of Food Allergy (SBAA).

## Results

Our patients were 48% male and 52 % female, and those numbers are not different from those of the pertinent literature. The age was distributed according with the literature, similar to all tree groups: 42.8% were infants, 32.7% were children (3 to 13 years) and 24.5% were adolescents and adults.

In anamneses of the GALT system, the abdominal pain was the most frequent complain, present in 15.6% of the patients, followed by diarrhea and vomits in 12.6 and constipation in 11.7%, next to them we had vomiting in 11.7% of patients studied.

In the physical examination of the patients selected to the study, pain of the right inferior abdômen was present in 18% of patients followed by abdominal distension in 13.5%. Left abdominal pain was found in 3.3% .Epigastric pain and splenomegaly in 2.6%.

## Conclusion

One explanation for the RLAP in patients with food allergy is related to the ileal nodular lymphoidhyperplasia expressing the immunereaction at the Payers Patches in the terminal ileum. Another splanation to the pain is the degranulation of eosinophils in the mucosal or muscular structure of the bowel wall inducing pain.

All physician doing physical examination in patients with the suspicious diagnosis of food allergy, have to do a careful examination of the right low abdomen, considering the presence of pain. The evidence of this signal will be very suggestive for food allergy.

